# Distinct evolutionary trajectories of V1R clades across mouse species

**DOI:** 10.1186/s12862-020-01662-z

**Published:** 2020-08-08

**Authors:** Caitlin H. Miller, Polly Campbell, Michael J. Sheehan

**Affiliations:** 1 Neurobiology and Behavior, Cornell University, Ithaca, USA; 2 Evolution, Ecology and Organismal Biology, University of California-Riverside, Riverside, USA

**Keywords:** V1R, Vomeronasal, Pheromone, Gene family, Gene expansion, Clade, *Mus*, House mouse

## Abstract

**Background:**

Many animals rely heavily on olfaction to navigate their environment. Among rodents, olfaction is crucial for a wide range of social behaviors. The vomeronasal olfactory system in particular plays an important role in mediating social communication, including the detection of pheromones and recognition signals. In this study we examine patterns of vomeronasal type-1 receptor (V1R) evolution in the house mouse and related species within the genus *Mus*. We report the extent of gene repertoire turnover and conservation among species and clades, as well as the prevalence of positive selection on gene sequences across the V1R tree. By exploring the evolution of these receptors, we provide insight into the functional roles of receptor subtypes as well as the dynamics of gene family evolution.

**Results:**

We generated transcriptomes from the vomeronasal organs of 5 *Mus* species, and produced high quality V1R repertoires for each species. We find that V1R clades in the house mouse and relatives exhibit distinct evolutionary trajectories. We identify putative species-specific gene expansions, including a large clade D expansion in the house mouse. While gene gains are abundant, we detect very few gene losses. We describe a novel V1R clade and highlight candidate receptors for future study. We find evidence for distinct evolutionary processes across different clades, from largescale turnover to highly conserved repertoires. Patterns of positive selection are similarly variable, as some clades exhibit abundant positive selection while others display high gene sequence conservation. Based on clade-level evolutionary patterns, we identify receptor families that are strong candidates for detecting social signals and predator cues. Our results reveal clades with receptors detecting female reproductive status are among the most conserved across species, suggesting an important role in V1R chemosensation.

**Conclusion:**

Analysis of clade-level evolution is critical for understanding species’ chemosensory adaptations. This study provides clear evidence that V1R clades are characterized by distinct evolutionary trajectories. As receptor evolution is shaped by ligand identity, these results provide a framework for examining the functional roles of receptors.

## Background

Olfaction involves detecting and discriminating among chemicals in the environment. Chemical compounds can vary considerably in structure, creating a highly complex chemical space in which olfactory systems evolve. In most mammals, olfaction relies on two discrete receptor systems: main olfactory receptors (ORs) and vomeronasal receptors (VRs) [1–3]. ORs detect a broad range of environmental odors [4–6], while VRs are integral to species-specific chemical detection, including pheromone detection [7, 8]. In humans, ORs are the only olfactory receptors, as the vomeronasal system is no longer functional. In other species, VRs mediate a wide range of social behaviors, including sexual, aggressive, and parental behaviors [9–18]. VRs thus provide a unique window into the chemical basis of social behaviors and the evolution of pheromone detection.

Across species, VRs exhibit striking evolutionary patterns. Whereas ORs have largely orthologous relationships among divergent species [19], VR evolution is characterized by rapid gene turnover wherein receptors are quickly gained and lost over evolutionary time [20–22]. This pattern of gene birth-and-death results in lineage-specific receptor repertoires [19]. Consequently, there are substantial differences in receptor sequences and repertoire size across divergent species [22–29]. For example, among three mammalian species (dog, opossum, and house mouse) there are virtually no one-to-one VR orthologs [19]. This is perhaps not surprising given the broad evolutionary timescale examined. However, even among two murine rodent species (the rat and house mouse), the majority of VRs fall into lineage-specific clades with very few orthologs [21, 24]. In addition to the evolutionary changes resulting from gene turnover, selection analyses on VRs across mouse species have revealed mixed results. Some studies find evidence for positive selection and lineage-specific pseudogenization [30, 31], while another detects evidence of genetic drift and negative selection [32].

As one of the leading model organisms, further understanding the evolution of chemosensation in the house mouse will provide insight into how chemical stimuli mediate distinct behavioral and neural responses. House mice are valuable models for examining VRs as they have large VR repertoires and there exists a wealth of knowledge on their social behavior, neural activity, and genetics [14–18, 33–39]. Currently, very few VRs have known ligands, which presents a significant barrier to studying the mechanisms underlying social behavior in house mice [33, 40–42]. By examining the evolutionary trajectories of VRs, we may uncover evolutionary patterns among receptor clades, and thereby identify targets for study based on the extent of turnover or conservation observed.

Vomeronasal sensory neurons express two major gene families in a cell-specific manner: V1Rs (type-1 VRs) and V2Rs (type-2 VRs) [4, 23]. V1Rs consist primarily of single-exon genes whereas V2Rs are multi-exonic [43]. Structurally, V1Rs have a short N-terminal extracellular region whereas V2Rs have long and highly variable N-terminal domains [4, 43]. We focus on V1Rs in this study due to the genetic tractability of their simpler gene structure for transcriptome assembly and sequence analysis. In functional terms, V1Rs primarily detect airborne volatiles [13, 14, 43–46]. In house mice, V1Rs have been implicated in detecting a wide range of volatiles, including urinary steroid molecules that are crucial for gender discrimination and sexual behaviors [40, 47–51].

**Fig. 1:**
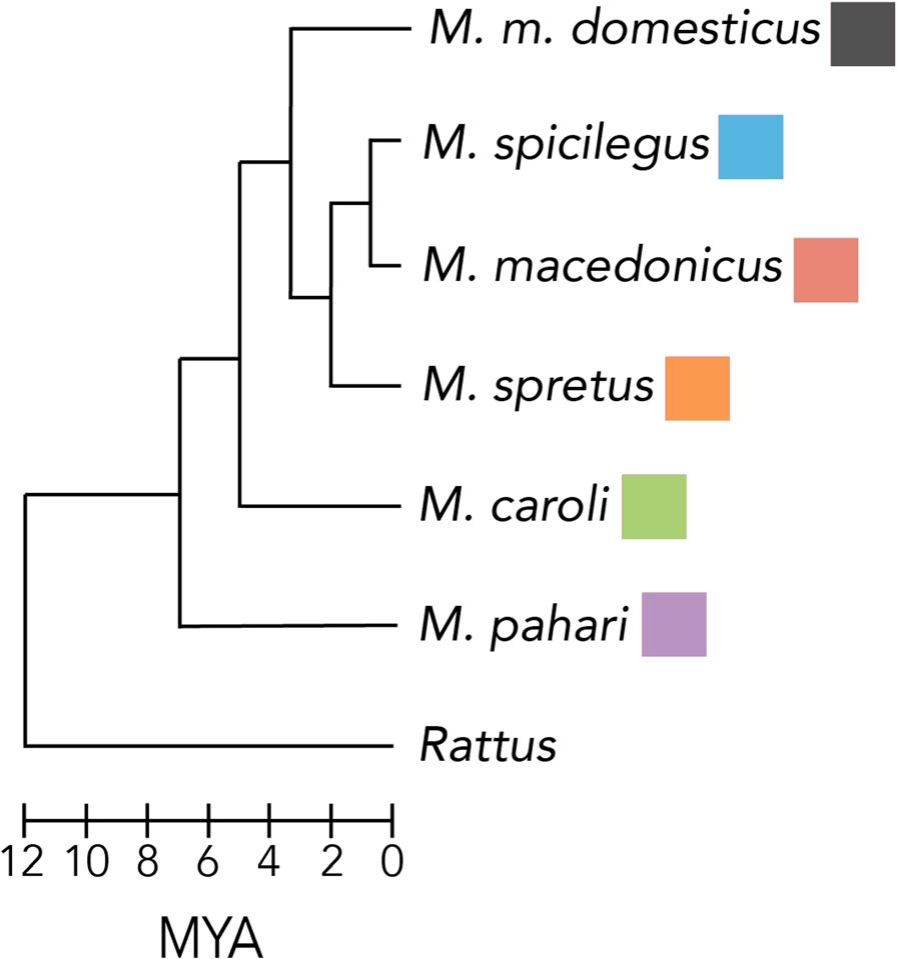
*Mus* species phylogeny. Includes all species in study [52, 53]. Rat (*Rattus norvegicus*) provided as outgroup. Species colors used throughout

Here, we characterize patterns of V1R evolution among the house mouse and relatives. We take a molecular evolutionary approach and analyze V1R repertoires across six species within the genus *Mus* (Fig. 1): *M. m. domesticus* (house mouse), *M. spicilegus*, *M. macedonicus*, *M. spretus*, *M. caroli*, and *M. pahari*. By examining the under-explored timescale of VR evolution among closely related species, this dataset offers new insight into the dynamics of VR evolution and provides a framework for understanding the selective pressures shaping V1R clades. Investigating the evolutionary history of V1R clades may in turn guide future efforts to deorphanize receptors in the house mouse, as the evolutionary trajectories of receptors are shaped by the ligands they detect. Ultimately, molecular evolutionary approaches to sensory gene repertoires seek to link function to evolutionary patterns [25, 26, 54–56]. For example, we can hypothesize that receptors detecting predator odors are highly conserved among mice due to shared or closely related predators among mouse species [57]. The present lack of resolved receptor-ligand relationships for most V1Rs precludes a comprehensive analysis of how patterns of gene turnover and selection regimes relate to ligands. The present work lays the foundation for such analyses in the future when more V1R ligands have been identified. In the present study, we provide detailed analyses of V1R clades known to detect estrus and pup cues in house mice [40, 42, 49].

## Results

### VNO sequencing, assembly & V1R recovery

Using wild-derived inbred mouse lines, we characterize V1R repertoires for five *Mus* species of varying evolutionary distance from the house mouse (1.5–7 mya, Fig. 1) by sequencing their VNO transcriptomes using short-read platforms. By sequencing both males and females from inbred mouse lines our aim was to characterize the V1R gene family for each species, rather than differential gene expression or within-species variability, and subsequently compare those data to the house mouse reference genome. The final transcriptome assemblies for each species are of good quality (Table 1). We detect approximately twice the number of V1Rs than are currently annotated in the genomes of *M. spretus*, *M. caroli*, and *M. pahari* and provide the first *M. macedonicus* V1R dataset (Table 1). The number of V1Rs identified in *M. spicilegus* is in good agreement with existing genome annotations (Table 1). For one species (*M. spretus*), the short-read sequencing was performed at greater depth, and an additional round of long-read sequencing was done. This allows us to examine the effectiveness of short versus long-read sequencing for assembling large and highly duplicated gene families such as V1Rs. The total number of assembled transcripts is greater for the *M. spretus* short-read dataset, as expected from greater sequencing depth (Table 1).

**Table 1:** VNO transcriptome assembly statistics, V1R transcript recovery and genome annotations

Species	Total Transcripts	Mean Length (bp)	N50	% with ORF^f^	Total V1R Transcripts	V1Rs With Unique Annotations	V1R Genes Detected (With Duplicates)	Genome Annotated V1Rs
*M. m. domesticus*^*a*^	–	–	–	–	263	–	208	208
*M. spicilegus*^*b*^	228,809	664	1632	43	122	105	119	120
*M. macedonicus*	255,395	649	1630	42	129	117	126	–
*M. spretus*^*c*^	1,181,673	688	1587	31	134 **(253)**	108 **(146)**	120 **(180)**	85
*M. caroli*^*d*^	384,865	460	1919	42	131	110	126	50
*M. pahari*^*e*^	450,181	305	3107	45	117	93	113	45

The mouse reference genome is shown for comparison (*M. m. domesticus*, top)^a^. Recovery estimates combining short and long-read datasets for *M. spretus* are indicated in bold. ^a^GRCm38.p6, ^b^GenBank accession#: QGOO00000000, ^c^SPRET_EIJ_v1.1, ^d^CAROLI_EIJ_v1.1, ^e^PAHARI_EIJ_v1.1, ^f^ORF: open reading frame

**Fig. 2:**
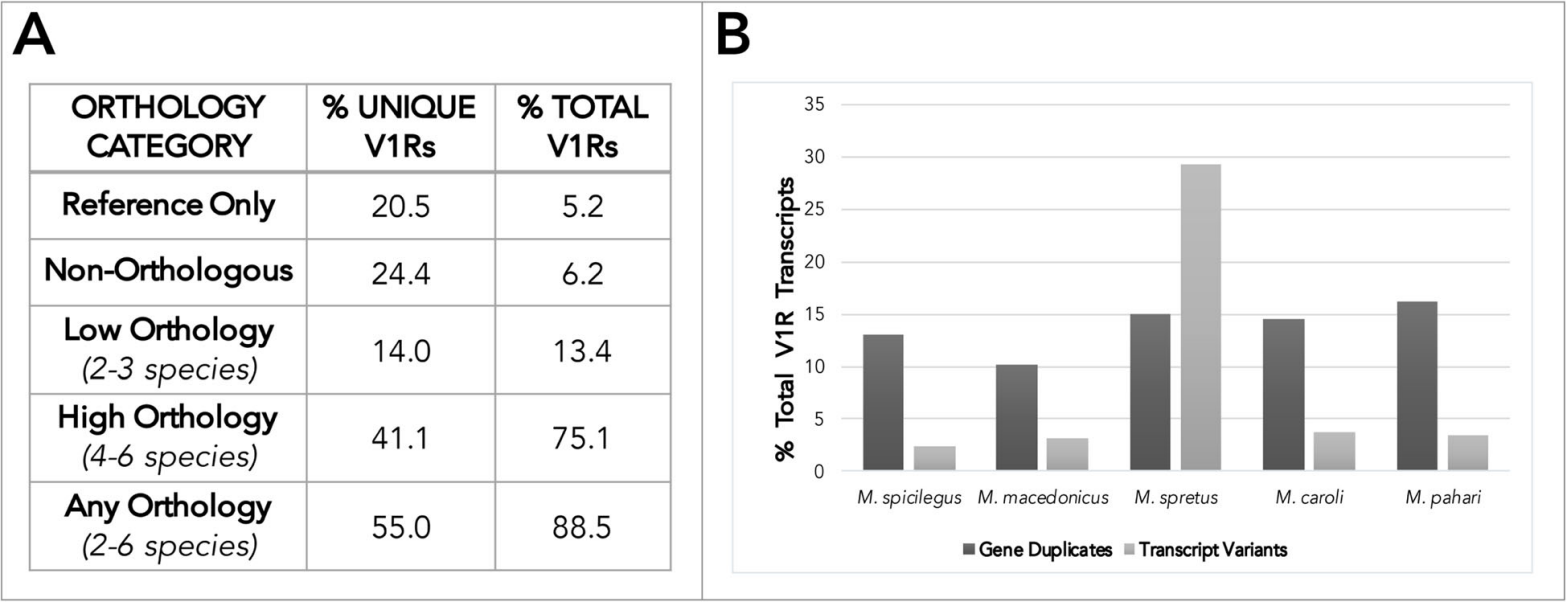
V1R orthology, gene duplicates, and transcript variants across *Mus* species. **a** The percent of unique and total V1Rs in each orthology category. **b** The percent of V1R transcripts that are either putative gene duplicates or transcript variants, for each species sequenced

On average, 126 V1R transcripts are recovered from each species’ short-read assembly (Table 1). A subset are transcript variants or gene duplicates, with homology to the same gene in the mouse reference genome (GRCm38.p6). The majority of V1Rs are single-exon genes, however, a substantial number contain introns and express transcript variants (Table 1 & Fig. 2) [38]. For a conservative estimate of V1R genes, only unique transcript annotations are included (Table 1). When putative gene duplicates are added, the number of V1R genes increases markedly (Table 1). Compared to the house mouse the 5 sequenced *Mus* species have smaller V1R repertoires, consistent with V1R gene expansion in the house mouse (Table 1). However, the addition of long-read sequencing for *M. spretus* increases the number of V1R genes detected, resulting in a repertoire size similar to the house mouse (Table 1). Therefore, whereas the *M. spretus* V1R repertoire is likely close to complete, long-read sequencing may detect additional V1Rs in *M. spicilegus, M. macedonicus, M. caroli* and *M. pahari*. Importantly, our analysis of V1R evolution in *Mus* is based on (1) a well-annotated mouse reference genome, (2) a comprehensive *M. spretus* V1R dataset, and (3) > 100 V1Rs for all 6 *Mus* species. Therefore, small gaps in detection across the entire V1R family should not bias the broad patterns of V1R evolution reported here. Furthermore, the discrepancy in repertoire size between the house mouse and other species appears largely accounted for by a putative house mouse specific gene expansion, discussed in further detail below.

### V1R evolution across *Mus* species

To explore V1R evolution, we characterize which receptors share a common ancestor (i.e. are orthologous) by examining relationships within a V1R gene tree containing six *Mus* species (the 5 sequenced species and the house mouse reference, Additional File 1). A subset of receptors does not exhibit a clear orthologous relationship to any V1R annotated in the mouse reference genome and are classified as non-orthologous genes, indicating either gene loss in the house mouse lineage or lineage-specific expansions in other species (Fig. 2). Similarly, a set of receptors annotated in the mouse reference genome are not detected in any other species, suggesting recent expansion in the house mouse lineage (Fig. 2).

We classify V1Rs into three broad categories based on their orthologous relationships: (1) V1Rs present only in the mouse reference genome*,* (2) non-orthologous V1Rs found in species other than the house mouse, and (3) V1Rs with orthology across multiple species. V1Rs with orthology across multiple species are further categorized based on the number of species represented in each orthologous receptor group (orthogroup). Orthogroups with 2–3 species are classified as “low orthology,” and orthogroups with 4–6 species as “high orthology” (Fig. 2a). The majority of transcripts have some evidence for orthology (88.5%, Fig. 2a). Furthermore, most transcripts are highly orthologous (75.1%, Fig. 2a), indicating that missing V1Rs are unlikely to bias broad patterns identified here. Although many receptors are shared across species, approximately 25% of all V1R transcripts, and 59% of all unique V1R annotations, are either low orthology, non-orthologous, or present only in the mouse reference genome (Fig. 2a). This indicates that the dramatic V1R gene turnover observed among more divergent mammalian species, such as across tetrapods or between rodent species [19, 21, 23], is replicated within the genus *Mus* albeit on a more limited scale. We further find a little over 5% of total V1Rs are present in only the house mouse reference genome. Nearly all of these reference-only receptors are located in a single clade and are tandemly arrayed on a single chromosome, suggesting a potential house mouse specific expansion.

We next examine the presence of gene duplicates and transcriptional variation across species (Additional File 2). A similar proportion of V1R gene duplicates are identified across all 5 species (10–16%, Fig. 2b). The proportion of V1R transcript variants detected is also comparable across species, with the clear exception of *M. spretus* (Fig. 2b). As expected, the addition of long-read (*M. spretus*) sequencing data recovers many more transcript variants than short-read sequencing datasets (Fig. 2b). Interestingly, the same number of V1R genes expressing distinct coding transcript variants are detected in *M. spretus* as in the house mouse (43 V1R genes, Additional File 3: Figure S1). However, the identity of V1Rs exhibiting alternative spliceforms, and the clades they belong to, vary between the two species (Additional File 3: Figure S1). In contrast, the proportion of gene duplicates detected is similar between *M. spretus* and the other species. This indicates that, for gene families such as V1Rs, short-read datasets are sufficient for identifying gene duplicates.

**Fig. 3:**
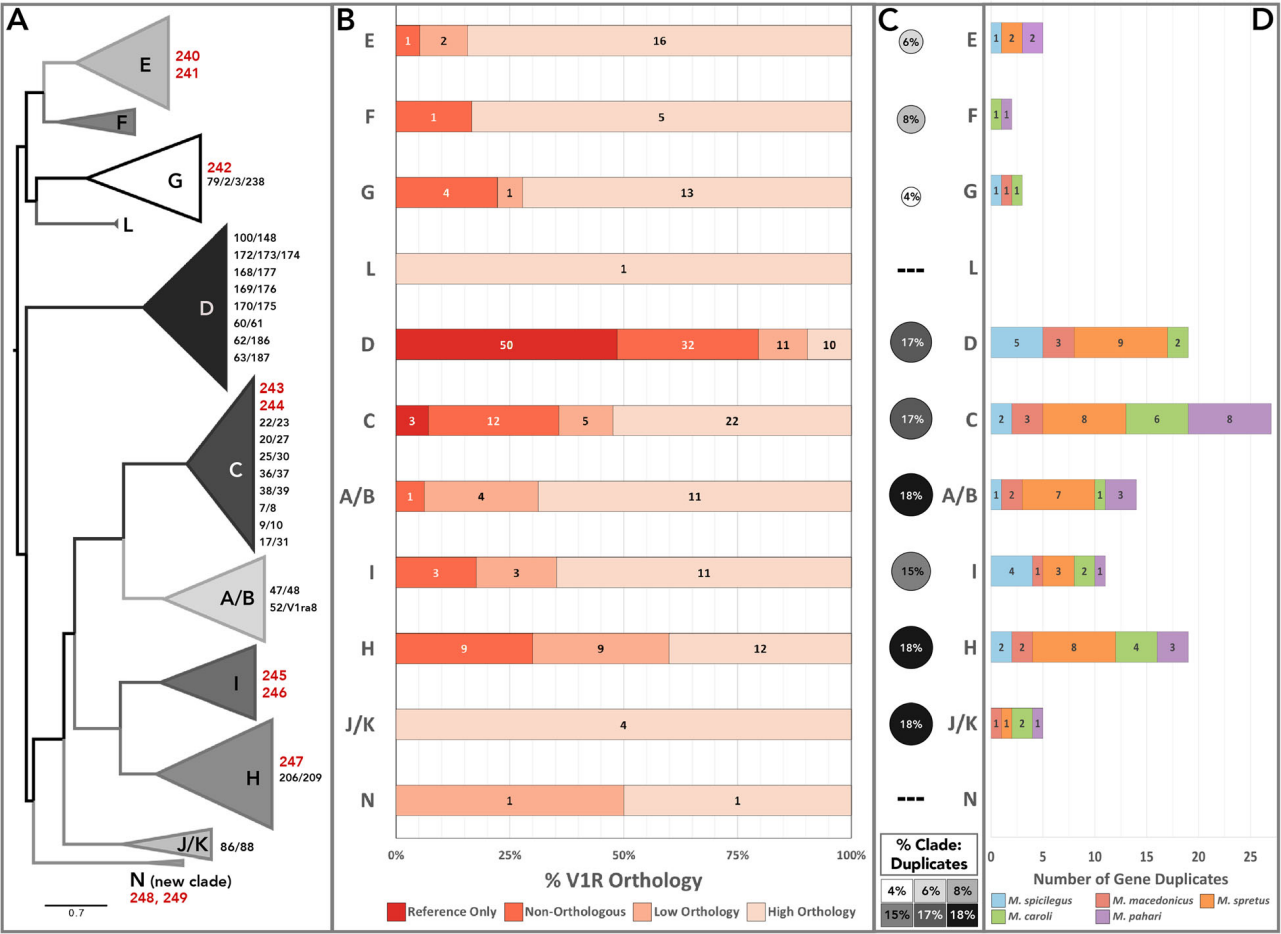
Patterns of orthology and gene duplication across V1R clades. **a** Phylogeny of all 11 V1R clades. Scale bar indicates 0.7 amino acid substitution per site. Shown to the right of each clade: V1R gene losses in the house mouse (red), and orthogroups with multiple reference annotations (combination-IDs, black). **b** Percent of V1Rs by clade that fall into each orthology category: reference only, non-orthologous, low orthology and high orthology. **c** Percent of total (sequenced) transcripts in each clade composed of gene duplicates (reference not included). The size and color of each circle corresponds to the calculated percentage. **d** Total number of V1R gene duplicates detected in each clade for each species

Our characterization of V1R repertoires across *Mus* species allows for a reliable estimate of V1R gene loss in the house mouse. We detect evidence for 10 such gene losses, distributed across six clades (Table 2 & Fig. 3a: indicated in red text). All V1R genes lost in the house mouse are present in at least 3 of the 5 sequenced *Mus* species, including close relatives (Table 2). Most gene losses have corresponding pseudogenes in the house mouse (Table 2). It appears gene losses are relatively uncommon compared to the abundant gene gains, at least within the house mouse lineage.

**Table 2:**
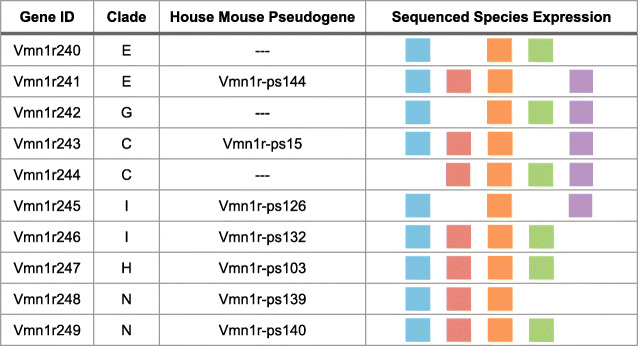
V1R gene losses in the house mouse

Species with receptor expression are indicated with different colors: *M. spicilegus* (blue), *M. macedonicus* (red), *M. spretus* (orange), *M. caroli* (green), and *M. pahari* (purple).

### Novel V1R clade: clade “N”

In addition to the house mouse gene losses observed in clades E, C, H, I and G, we identify a novel V1R clade (Table 2, Fig. 3a). This novel clade “N” has been lost in the house mouse and consists of two receptor orthogroups. Both clade N receptors (*Vmn1r248* and *Vmn1r249*) are expressed in at least three *Mus* species (Additional File 3: Figure S2) and have corresponding pseudogenes in both the house mouse (*M. m. domesticus*) and the rat (*Rattus norvegicus*).

### Variable patterns of evolution across V1R clades

#### Gene turnover: orthology, duplication & repertoire size

The maintenance or loss of gene orthologs is a major mode of chemosensory evolution [19, 21, 23, 27]. If different clades exhibit either high degrees of gene orthology or lineage-specific gene expansions, this suggests distinct evolutionary trajectories. Patterns of V1R gene orthology and duplication vary across clades. Four clades are very orthologous (E, F, J/K and L: > 80% of receptors are high-orthology), with clade G trailing behind with more non-orthologous receptors (Fig. 3a, b). Each of these clades has 5 or fewer gene duplicates, however, the proportion of duplicates is variable (Fig. 3c, d). Clades E, F and G have very low proportions of gene duplicates, while clade J/K has among the highest (Fig. 3c).

Clades C, D and H have abundant low-orthology and non-orthologous receptors (Fig. 3b), indicating greater evolutionary lability. While most orthologous relationships are straightforward, some orthogroups contain multiple house mouse receptors, and are annotated with combination-IDs to indicate the relationship to multiple genes (e.g. *Vmn1r25/30*). These receptor groups are the result of one or more duplication events within the *Mus* lineage, and are unequally distributed across clades, with 76% located in clades C and D (Fig. 3a). In addition, all reference-only V1Rs are located in these same two clades (Fig. 3b). Not surprisingly, clades C, D and H have the highest number of detected gene duplicates (19 or more) and have similarly high proportions of duplicates by clade size (Fig. 3c, d). Thus, all three clades have evidence for substantial gene expansions, particularly clade D within the house mouse lineage.

**Fig. 4:**
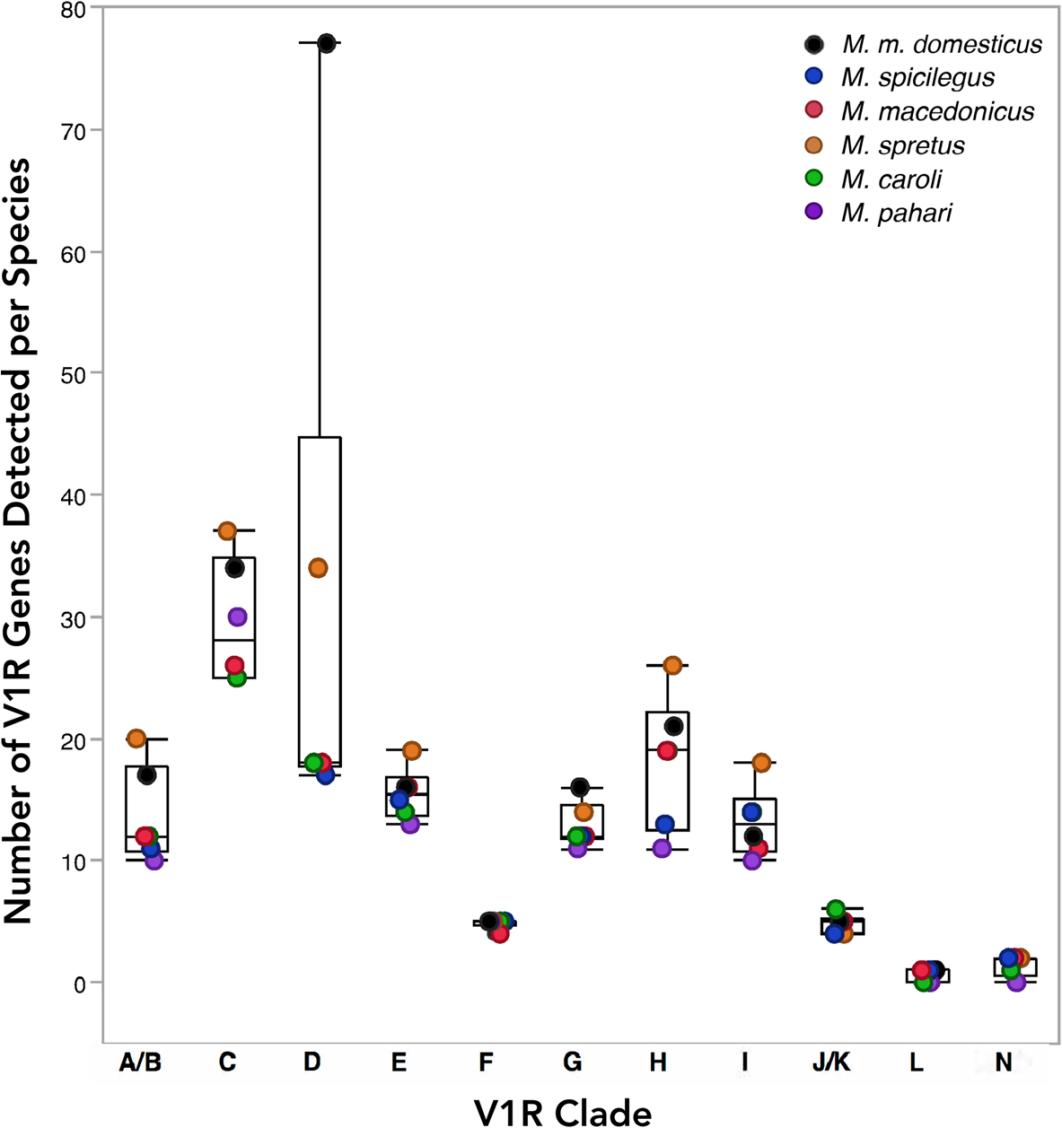
V1R clade receptor repertoire sizes across *Mus* species. *Mus* species are indicated with different colors

We examine V1R clade sizes across all 6 species. With the striking exception of clade D, the house mouse clade sizes are very similar to the 5 other species, (Fig. 4). This general pattern provides further evidence that receptor recovery is high and species’ repertoires are near complete. Interestingly, the *M. spretus* repertoire is largest for several clades (A/B, C, E, H and I; Fig. 4), indicative of *M. spretus*-specific gene expansions.

The size ranges of two clades (A/B and D) are skewed by the house mouse and *M. spretus* datasets. Both species have much larger clade D repertoires than the other 4 species, exposing this clade as a potential hotspot for recent gene duplications (Fig. 4). On the other hand, the discrepancy in clade D repertoire size may be the result of poor receptor recovery for this clade, such that additional long-read sequencing may reveal comparable patterns in other species. An existing VR expression dataset sheds some light on this, as it finds clade D receptors in house mice are more lowly expressed than other clades [38]. However, while the *M. spretus* clade D is larger than the other sequenced mouse species, it is still considerably smaller than the house mouse (43 fewer receptors, Fig. 4). Therefore, despite potential low clade D receptor recovery, our data still suggest a large house mouse specific clade D expansion. In contrast, there are several other clades which exhibit low variation in repertoire size across all species’ datasets (E, F, G, J/K, L, N). Furthermore, clades C and H display variation in repertoire size across all 6 species, providing evidence for species-specific V1R gains and losses in multiple lineages (Fig. 4).

#### Patterns of positive selection

To further examine the selective pressures shaping V1R clade evolution, we tested orthogroups (with at least four orthologs and/or paralogs) for the presence of episodic positive selection using an adaptive branch-site random effects model based on dN/dS estimates [58]. A total of 127 orthogroups containing 685 V1R genes (including putative gene duplicates) were tested across all 11 clades (Table 3). We find evidence for 24 V1R genes under positive selection (3.5% of genes tested), as well as 8 deeper branches (2.3% of deeper branches tested) under a 5% false discovery rate (FDR) (Table 3). There are no noticeable differences in the number of branches under positive selection across species (Additional File 3: Table S1), however, some striking differences exist across clades (Table 3). The stark patterns of gene turnover evidenced by gene orthology and duplication, do not always align with positive selection trends. This may be due to the fact that high gene turnover makes detecting positive selection more challenging. However, it may also suggest that different clades are experiencing distinct diversifying selective pressures, ranging from large-scale gene gains and losses, to small-scale receptor sequence divergence. The most striking example is clade G, which is very orthologous with extremely low rates of gene duplication (Fig. 3), but simultaneously sports almost double the percentage of branches under positive selection compared to other clades (Table 3). In contrast, clade D shows pronounced patterns of rapid evolutionary change, with evidence for gene turnover (particularly gene gains in the house mouse lineage) as well as a large number of genes under positive selection (Table 3). Similarly, clade E demonstrates a consistent pattern of conservation with minimal gene turnover, and the lowest proportion of branches under positive selection (Table 3).

**Table 3:** V1R branches under positive selection across clades

CLADE	TERMINAL Branches^a^	INTERNAL Branches^b^	TOTAL Branches	TESTED Branches	% Branches^c^	TESTED Orthogroups	% Orthogroups^d^	Orthogroup IDs^e^
A/B	3	2	5	**119**	4.20	**11**	27.27	Vmn1r45, Vmn1r47/48, Vmn1r52
C	3	2	5	**235**	2.13	**35**	11.43	Vmn1r11, Vmn1r21, Vmn1r25/30, Vmn1r38/39
D	5	0	5	**119**	4.20	**13**	30.77	Vmn1r60/61, Vmn1r172/173/174, Vmn1r183, V1rd19
E	0	1	1	**122**	0.82	**16**	6.25	Vmn1r241
F	2	0	2	**45**	4.44	**5**	20.00	Vmn1r235
G	6	2	8	**101**	7.92	**13**	38.46	Vmn1r74, Vmn1r76, Vmn1r81, Vmn1r83, Vmn1r242
H	3	0	3	**125**	2.40	**15**	20.00	Vmn1r205, Vmn1r206/209, Vmn1r247
I	2	0	2	**100**	2.00	**13**	15.38	Vmn1r192, Vmn1r193
J/K	0	1	1	**57**	1.79	**4**	25.00	Vmn1r85
L	0	0	0	**7**	0.00	**1**	0.00	–
N	0	0	0	**5**	0.00	**1**	0.00	–
Total	24	8	32	1034	3.09	127	19.05	NA

The total number of tested branches and orthogroups are bolded, all other data columns correspond to branches under positive selection (*P* ≤ 0.05). All branches, including counts and percentages, are after 5% FDR correction. ^a^Terminal branches correspond to genes. ^b^Internal branches correspond to deeper branches in the orthogroup. ^c^Percentage of total branches tested in a given clade under positive selection. ^d^Percentage of the total number of orthogroups tested in a given clade with evidence for positive selection. ^e^Orthogroup gene IDs that contain at least one branch under positive selection.

Guided by the evolutionary patterns observed across clades, we identify and categorize receptors as interesting candidates for further functional work based on striking patterns of conservation or divergence (Additional File 3: Table S2). We hope this list will help guide future efforts to deorphanize V1Rs.

### Fast-evolving clades

#### Clade H

**Fig. 5:**
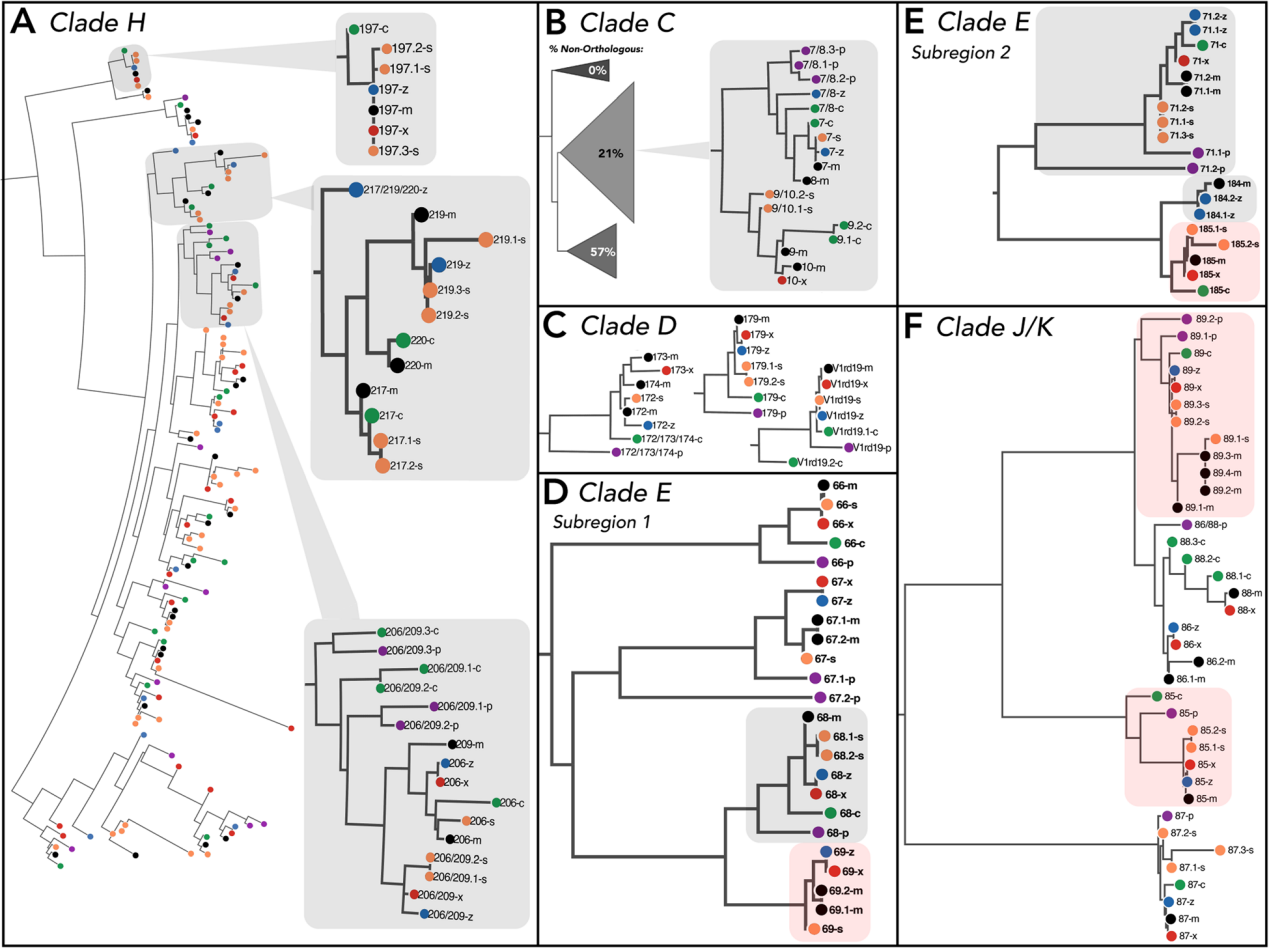
Example receptor groups depicting patterns of lineage-specific evolution and conservation across clades. V1R annotations are abbreviated (e.g. *Vmn1r137* as 137). Species transcripts annotated with the same reference gene have unique transcript IDs (e.g. 217.1 and 217.2). *Mus* species are indicated with colors and letters (*M. m. domesticus*: “m” and black; *M. spicilegus*: “z” and blue; *M. macedonicus*: “x” and red; *M. spretus*: “s” and orange; *M. caroli*: “c” and green; *M. pahari*: “p” and purple). **a** Clade H: entire clade shown, highlighting receptor groups that depict patterns of conservation and divergence. **b** Clade C: sub-clades shown with percentages of non-orthologous receptors. *Vmn1r7/8* and *Vmn1r9/10* are shown in detail. **c** Clade D: specific clade D receptor groups uniquely conserved across species. **d-e** Clade E: Specific receptor subregions of clade E. Highlighted receptors have some functional evidence (grey) or are deorphanized with strong evidence (red). **f** Clade J/K: entire clade depicted, highlighting specific deorphanized receptor groups that detect estrus cues (sulfated estrogens) in female urine

Clade H appears to be a mouse-specific V1R expansion, as it is absent in the rat genome [21]. The clade is characterized by low orthology, abundant gene duplicates, and variable repertoire size across species (Figs. 3 & 4). In contrast to the patterns of high gene turnover, relatively few clade H branches have evidence for positive selection (Table 3). A sub-region of clade H containing *Vmn1r217*, *219* and *220* receptors exemplifies this pattern of low orthology, while the receptor orthogroup *Vmn1r206/209* is representative of the abundant gene duplicates (Fig. 5a). Intriguingly, the *Vmn1r206/209* orthogroup also has evidence for positive selection, pointing to strong diversifying selection within this receptor group (Table 3). A striking exception to the evolutionary lability of clade H is the highly conserved *Vmn1r197* receptor group (Fig. 5a). The general pattern of rapid species-specific gene gains and losses suggests clade H receptors may play an important role in detecting complex species-specific signals.

#### Clade C

Clade C is the largest V1R clade across species with the exception of the house mouse. Clade C also exhibits variable repertoire sizes across species, indicative of lineage-specific evolution (Fig. 4). This inference is supported by the large numbers of combination-ID orthogroups, gene duplicates, non-orthologous receptors, and house mouse-specific gene gains (Fig. 3). In contrast to the high levels of gene turnover, relatively few clade C branches have evidence of positive selection (Table 3). The phylogenetic structure of clade C comprises three sub-clades, one of which is quite orthologous (Fig. 5b). Non-orthologous receptors are largely clustered in one subclade (57%, Fig. 5b), while the majority of receptors under positive selection are located in another (21%, Fig. 5b). Together, this suggests these subclades may be experiencing distinct forms and rates of receptor evolution. Two clade C receptors, *Vmn1r9* and *Vmn1r10*, have been implicated in pup odor detection in house mice [42]. However, these receptors also respond to female odors, and may detect chemosensory components of the nest environment [42]. These two receptors are part of a single receptor orthogroup (*Vmn1r9/10*) that is both orthologous and highly duplicated (Fig. 5b). The sister group *Vmn1r7/8* exhibits a similar pattern of high orthology and abundant duplication (Fig. 5b). Given the potential role of *Vmn1r9/10* receptors in pup odor detection, and the lineage-specific evolutionary patterns observed in *Vmn1r7/8* and *Vmn1r9/10,* these receptor groups are interesting candidates for future functional tests of their role in conspecific chemosignaling.

#### Clade D

Clade D exhibits a large skew in repertoire size within the house mouse (Fig. 4), and has the most dramatic pattern of non-orthology across all V1R clades (Fig. 3b). Nearly all reference-only V1Rs (50/53: 94%) are located in clade D, providing further support for a large recent gene expansion in the house mouse, despite the potentially low receptor recovery of other species in this clade (Fig. 3b). These receptors are similar in sequence and cluster together on chromosome 7, consistent with recent tandem gene duplication. While we do not find evidence for a comparably large expansion in the other mouse species, we recover approximately twice as many clade D receptors in *M. spretus* relative to the other four species (Fig. 4). It is possible that similar expansions exist in the other species that are not detected here, particularly given prior evidence that clade D receptors are lowly expressed in the house mouse [38]. Clade D has a high proportion of non-orthologous receptors and gene duplicates, as well as a large percentage of orthogroups under positive selection (30.77% of tested orthogroups, Table 3). Given the evolutionary lability of clade D, there are a few receptors that stand out as highly orthologous (*V1rd19*, *Vmn1r179* and *Vmn1r172/173/174,* Fig. 5c), two of which have evidence for positive selection (Table 3). Clade D appears to be experiencing lineage-specific evolution at the scale of both gene gains and losses, as well as sequence divergence.

### Conserved clades & female conspecific detection

#### Clades E & F

A subset of V1R clades are highly conserved, and thus good targets for uncovering receptors with conserved olfactory functions. Clades E and F are characterized by high orthology (Fig. 3b), long internal branch lengths and short terminal branch lengths, suggestive of old gene duplications maintained within the *Mus* lineage (Additional File 3: Figure S3). In contrast, very few recent gene duplications are detected (Fig. 3c, d). In addition, clade E has the lowest proportion of branches under positive selection of any clade (Table 3). Clade F, on the other hand, has a single receptor (*Vmn1r235*) with evidence for positive selection in two species (Table 3, Additional File 3: Table S1). A subset of 5 clade E receptors is important for the detection of female-specific urine odors in house mice (*Vmn1r68, Vmn1r69, Vmn1r71, Vmn1r184, Vmn1r185*) [40]. Two clade E sub-regions containing these same 5 receptor groups are shown in Fig. 5d, e; those with the strongest support for female odor detection are highlighted in red (*Vmn1r69* and *Vmn1r185*) [40]. *Vmn1r68* and *Vmn1r69* are sister to each other in the gene tree and are highly orthologous, however, *Vmn1r69* has no orthologs detected among the more basal species (*M. caroli* and *M. pahari*; Fig. 5d). It is plausible that *Vmn1r69* is the result of a gene duplication event preceding the divergence of the four more derived species (Fig. 5d), providing enhanced specificity or sensitivity toward female-specific urine odors. The second clade E sub-region contains receptors: *Vmn1r184*, *Vmn1r185,* and *Vmn1r71*. *Vmn1r184* and *Vmn1r185* are sister receptor groups, in which *Vmn1r185* is highly orthologous and *Vmn1r184* appears to be the result of a recent duplication event (Fig. 5e). *Vmn1r184* is detected in only the house mouse and *M. spicilegus* (Fig. 5e). Furthermore, *M. spicilegus* has evidence for a species-specific *Vmn1r184* duplicate, and has an absence of *Vmn1r185* expression (Fig. 5e). The distinct expression pattern of *Vmn1r184* in *M. spicilegus* is noteworthy given this species’ unique social structure, which includes cooperative behaviors and social monogamy [59]. In comparison, *Vmn1r71* is highly orthologous (Fig. 5E), but displays remarkable transcriptional variability, most of which is located at either the C-terminus or N-terminus regions of the protein (Additional File 3: Figure S3). Broadly, clades E and F display patterns of conservation, with some evidence of positive selection in clade F, and potential lineage-specific gains in clade E among receptors involved in detecting female cues.

### Clade J/K evolution & the detection of estrus cues

Clade J/K is a small clade of only 4 receptor groups that is highly orthologous and boasts one of the highest proportions of gene duplicates (Fig. 3). This clade thus encompasses a unique mixture of conservation and expansion, in which there is very little gene loss but gene gains are abundant (Figs. 3 & 5f). Clade J/K is also the only clade for which half of the receptors have known ligands [40, 49]. In the house mouse, two of the four J/K receptors have been shown to detect estrus cues (i.e. sulfated estrogens) in female urine [40, 49]. Given the unique features of this clade, we examined in greater detail the amino acid changes across species within the two deorphanized receptors (*Vmn1r85* and *Vmn1r89*). The *Vmn1r89* receptor group has evidence for short and long transcript types across *Mus* species (Additional File 3: Figure S4). Many species have only one form detected. However, the house mouse and *M. spretus* express both forms as transcript variants, while *M. pahari* appears to have distinct genes generating these two forms (Additional File 3: Figure S4). The widespread detection of both transcript types suggests they may facilitate the detection of distinct ligand (i.e. sulfated estrogen) features. This is particularly compelling given that in the house mouse, *Vmn1r89*-expressing VSNs detect multiple sulfated estrogen molecules and are more broadly tuned than *Vmn1r85-*expressing VSNs [40]. In comparison, the *Vmn1r85* receptor group is highly conserved among the 3 *Mus* species most closely related to the house mouse (Fig. 5f), with the majority of substitutions concentrated in *M. caroli* and *M. pahari* (Additional File 3: Figure S5). For both *Vmn1r85* and *Vmn1r89,* the highest proportion of amino acid site changes detected across species occurs in extracellular regions (Additional file 3: Figure S6). The trend towards greater extracellular substitutions is consistent with a prior analysis of molecular evolution in 22 V1Rs, demonstrating that most sites with evidence for positive selection are located in extracellular motifs [30]. Moreover, positive selection is detected in *Vmn1r85* (Table 3) at an internal branch containing the house mouse and close relatives (*M. spicilegus, M. macedonicus* and *M. spretus,* Additional File 3: Table S1).

## Discussion

### V1R clades are characterized by distinct evolutionary trajectories

The complexity of the chemical environment presents unique evolutionary challenges. In addition to detecting a vast range of chemical stimuli, olfactory systems must flexibly adapt to novel environments and social contexts. One of the primary mechanisms of chemoreceptor evolution is through gene birth-and-death, mediated by duplication events and pseudogenization [21, 23, 27]. Across divergent mammalian species, VRs have been shown to be fast-evolving with high gene turnover and lineage-specific clades, compared to the more conserved and largely orthologous ORs [19]. This has led to the hypothesis that ORs are broadly-tuned generalists, and VRs are more narrowly-tuned specialists [19]. Furthermore, olfactory specialization is hypothesized to occur through selection on distinct receptor subfamilies [23]. In this manner, receptor families may expand or contract in a lineage-specific fashion, and receptors in each family may become more diverse or conserved. Here, we identify distinct patterns of evolution among *Mus* V1R clades, consistent with a model of subfamily-specific selection. Some V1R clades have evidence of high gene turnover, while others are highly orthologous across species. We similarly detect variable patterns of positive selection across clades. Thus, the evolutionary patterns of gene turnover and positive selection are not always coincident, suggesting different evolutionary forces may act on clades in a distinct fashion. Furthermore, the evolutionary trajectories of clades could be driven by genomic processes such as variation in recombination rates across the genome. Future work examining V1R evolution in relation to additional features of the genome will be informative. Prior research has generated controversy over what evolutionary forces mediate V1R evolution. Some studies detect evidence of positive selection and lineage-specific pseudogenization, while another study finds evidence for genetic drift and negative selection [30–32]. Our data suggest that these seemingly contradictory results are not mutually exclusive. Depending on the subfamily of receptors examined, one could detect very different evolutionary patterns. This creates a functional framework in which to examine subsets of V1Rs, as receptor evolution is sculpted by the identity of their ligands.

### V1R gene gains and losses

Our results support the gene birth-and-death model of V1R evolution, exemplified by the variable patterns of orthology, gene duplicates, and sequence diversity observed across clades. However, while gene gains appear abundant across *Mus* species, clear evidence of gene losses are infrequent. A reliable estimate of V1R gene loss is restricted to the house mouse, due to constraints of V1R recovery among the other *Mus* species sequenced. Nevertheless, across all V1R clades only 10 gene losses are detected in the house mouse. We also identify a novel clade of two receptor groups, which appears to have undergone pseudogenization in house mice and in rats. This stands in contrast to a previous study examining the microevolution of V1Rs among *Mus musculus* subspecies, which detected a high frequency of null alleles [32]. On the other hand, functional gene duplicates appear plentiful. The most striking example is in the house mouse, in which clade D appears to have undergone a large species-specific gene expansion. As house mice successfully inhabit both commensal and non-commensal environments, it is tempting to posit that the clade D expansion may reflect a chemosensory adaptation to accommodate their expanded chemical environment [60, 61]. Commensal behavior would have originated (at the earliest) in conjunction with agriculture and permanent human settlements roughly 10,000 years ago. Thus, gene expansions likely predate commensal behavior and could plausibly facilitate the invasion of novel niches rather than an adaptation to it [62]. Overall, the abundant gene gains suggest that in the *Mus* genus, or at the very least within *Mus musculus* subspecies, expansion of the V1R family is ongoing.

### Patterns of receptor evolution and function

Only a handful of V1Rs have known ligands. However, it has become increasingly clear that a critical function of the VNO involves detecting heterospecific odors, such as predator cues [49, 57, 63, 64]. V1Rs tuned to detecting broad classes of predator cues (e.g. birds of prey, snakes or mammals) may be conserved across mouse species. In particular, clade F has been implicated in detecting mammalian predator cues [49]. The broad-scale patterns of conservation observed in clade F are consistent with the maintenance of a similar key function, such as the detection of predator odor cues with shared ligands [57]. Given the possible role of clade F in predator detection, further investigation of the sole receptor group (*Vmn1r235*) with evidence of positive selection warrants further investigation.

Chemical signaling is critical to social and reproductive interactions across a wide variety of mammalian species, including mice. One of the best described olfactory communication systems exists in house mouse urine scent marks [65]. House mice secrete proteins in their urine (major urinary proteins, MUPs) that facilitate pheromonal communication and individual recognition [17, 62, 66–70]. MUPs act as transport vessels for the slow-release of volatile compounds detected by V1Rs [8, 51, 66, 70]. As these protein ligands vary considerably across *Mus* species, their corresponding volatiles likely shift as well [66, 70]. As a result, clades such as C, D and H, exhibiting highly species-specific evolution may be good targets for the detection of social cues.

Mounting evidence suggests that V1Rs are crucial for detecting sex-specific cues and the physiological status of conspecifics [40, 48, 49, 71]. A subset of clade E receptors respond to female-specific urine ligands, as such, clade E conservation may be tied to detecting conspecific sex cues [40]. Clade D has also been implicated in detecting female odors [49]. However, the activation of clade D is quite specific to *Vmn1r167* [49]. Interestingly, *Vmn1r167* contains one of the largest species-specific (*M. spicilegus*) gene duplications, and is only detected in *M. spicilegus* and the house mouse. *Vmn1r167* may thus play an important derived role in female odor detection.

Previous work demonstrates that V1Rs are strongly activated by sulfated steroids, and up to 80% of ligands detected in female urine may be sulfated steroids [48]. Clade J/K has been shown to play an important role in detecting sulfated estrogen molecules [40]. As such, the pattern of conserved orthology in clade J/K may reflect a crucial role for these receptors in discerning information about the internal state of conspecifics, particularly female reproductive state. Furthermore, the proportionally high levels of positive selection and gene duplication suggests lineage-specific evolution is occurring, though maintaining receptor functionality is important.

## Conclusions

Understanding the evolutionary dynamics of the vomeronasal system reveals important properties of chemosensory evolution, as well as the functional roles of different receptors. In generating near-complete V1R repertoires for 5 *Mus* species, we find evidence for previously described patterns of high gene turnover observed among divergent species. However, by examining the evolutionary relationships of V1Rs across the *Mus* genus, we find that distinct receptor lineages have experienced different evolutionary trajectories, both at the level of gene gains and losses as well as sequence divergence. Thus, clade-level evolution is critical to understanding the chemosensory adaptations of species to their diverse chemical environments. Furthermore, the evolutionary patterns of V1Rs observed supports the proposition that the detection of physiological status and female-specific cues may be an important role of V1R chemosensation [40, 48, 49, 71]. Ultimately, these results provide a key foundation for future functional studies of V1Rs.

## Methods

### Animal strains and tissues

All mice sequenced in this study are from wild-derived inbred lines. Mouse strains for *M. caroli* (CAR: RBRC00823) and *M. spicilegus* (ZBN/Ms.: RBRC00661) were obtained from RIKEN BioResource Center (Japan). *M. pahari* (PAH/EiJ) was obtained from The Jackson Laboratory (Bar Harbor, ME). All strains were maintained in an Animal Care facility at Cornell University with a 14:10 shifted light:dark cycle, and provided food and water ad libitum. Experimental protocols were approved by the Institutional Animal Care and Use Committee (IACUC: Protocol #2015–0060), and were in compliance with the NIH Guide for Care and Use of Animals. All experimental mice were sacrificed by cervical dislocation, and the VNOs subsequently dissected. VNOs (stored in RNALater) for *M. macedonicus* (XBS) and *M. spretus* (SFM) were obtained from the Campbell Lab at Oklahoma State University (OSU). Mice at OSU were maintained on a 12:12 light:dark cycle and provided with food and water ad libitum. Live animal work at OSU was approved by the IACUC under protocol # AS-1-41.

### Illumina RNA library preparation & sequencing

VNO epithelia were dissected from at least one male and one female from each inbred wild-derived species line and subsequently pooled to obtain V1R repertoires unbiased to a particular sex, except for the HiSeq dataset (ZRU: 2 males, 2 females; XBS: 1 male, 1 female; CAR: 2 males, 2 females; PAH 1 male, 1 female; SFM short-read HiSeq: 0 males, 15 females; SFM long-read Isoseq: 1 male, 1 female). Variation in the number of individuals sampled per species was due to sample and data availability as well as dissection quality. This negligibly impacts our results as we are examining V1R repertoires not expression levels. Total RNA was extracted from VNO tissues using the Qiagen RNeasy kit, and subsequently quantified using QuBit Fluorometric Quantitation. RNA sequencing libraries were generated using the NEBNext Ultra RNA Library Prep Kit for Illumina (NEB #E7530). NEBNext Poly(A) mRNA Magnetic Isolation Module (NEB #E7490) was used for RNA Isolation. Sequences were indexed using the NEBNext Multiplex Oligos for Illumina (Dual Index Primers Set 1, NEB #E7600). A series of sequencing runs were performed on Illumina and Pacific Biosciences platforms. The VNO libraries for strains ZRU, XBS and CAR were sequenced as 300 bp paired-end reads on Illumina MiSeq platform through the Biotechnology Resource Center (Institute of Biotechnology) at Cornell University. Additional VNO RNA libraries for strains ZRU, XBS, CAR and PAH were sequenced as 150 bp paired-end reads on Illumina NextSeq 500 platform through the Biotechnology Resource Center (Institute of Biotechnology) at Cornell University. A series of 15 SFM female VNO samples were sequenced as 125 bp paired-end reads on Illumina HiSeq 2500 platform at Novogene (Sacramento, CA). Pacific Biosciences (PacBio) Isoseq libraries were also generated and sequenced from pooled SFM male and female VNOs. This additional long-read dataset ensured the *M. spretus* (SFM) species-wide V1R repertoire was captured, and allowed for insight into the effectiveness of short and long-read datasets for V1R detection.

### Transcript processing and assembly

FastQC reports were generated for each sample to ensure sequencing quality [72]. Trimmomatic was used to clean the raw reads [73]. The trimmed read files were concatenated for each species across the different Illumina sequencing runs. rnaSPAdes was used to generate de novo transcriptome assemblies for each species’ concatenated RNA sequencing dataset [74]. Transrate and rnaQUAST were used for assembly quality assessment [75, 76]. Other assemblers were tested (e.g. Trinity), however rnaSPAdes consistently assembled longer reads and more VRs were recovered from these assemblies.

### Isoseq library preparation and consensus assemblies

Pacific Biosciences Isoseq was used to generate long-read sequences for the VNO from *Mus spretus* at the Arizona Genome Institute. We sequenced 4 different library sizes 0.8–1.6 kb (× 3 smartcells), 1.3–2.6 kb (× 2 smartcells), 2.2–3.7 kb (× 2 smartcells) and > 3.0 kb (× 2 smartcells) generating a total of 19GB of raw data. These data were run through the PacBio smrtpipe version 2.3 by the Arizona Genome Institute, generating polished high consensus sequences, which we analyzed further for V1R genes.

### Identification of V1R sequences

The Ensembl reference annotation (version 94) of the mouse reference genome (GRCm38.p6) was used to download all known sequences for V1Rs. These reference sequences were used in a series homology-based searches (blastn, blastx and tblastn) to identify putative V1Rs in the RNA transcript assemblies for each mouse species. GetORF [77] was then used to identify open reading frames (ORFs) among the putative V1R dataset, using a well-defined V1R gene model [38]. Dedupe was used to remove exact duplicate DNA sequences and containment DNA sequences within this refined ORF dataset. DNA sequences were translated into corresponding peptide sequences using GetORF [77]. MAFFT v. 7 was used to align the peptide V1R sequences for each species, and sequences with less than 30% identity with the entire V1R group for a given species were eliminated from further analysis [78]. While this pipeline was designed to identify functional V1R genes, given the abundance of V1R pseudogenes and the incomplete genome annotations for many of these species, some pseudogenes may inadvertently be included in these analyses.

### V1R annotation and identification of orthologous receptors

Putative V1Rs were first annotated based on homology to the mouse reference genome. If multiple transcripts were *most similar* to a specific reference V1R gene (e.g., *Vmn1r30*), these transcripts were annotated with this same gene ID, and distinguished with unique numbers following the gene ID (e.g., *Vmn1r30.1* and *Vmn1r30.2*). Some annotations based on homology and their orientation within the gene tree did not always perfectly match due to the effects of gene duplications and losses at varying points in the *Mus* phylogeny. As such, some V1R annotations were adjusted upon analysis of the phylogenetic relationships of receptor sequences within the maximum-likelihood gene tree. The most important criteria for determining orthologous receptor groups was the relative orientation of all 6 species, under the general rule that the receptor phylogeny should recapitulate the species phylogeny. Using the annotation system of the reference genome meant that some gene duplications with distinct reference annotations were included in the same receptor ortholog group. Thus, some orthologous receptor groups were annotated with combination-IDs (e.g. *Vmn1r25/30*). Furthermore, a proportion of receptors from each of the five sequenced non-reference species were non-orthologous in that they did not fall into any particular ortholog group, but were basal to multiple groups or to several reference genes. These non-orthologous receptors were annotated based on the genes they were basal to, either as a combination-ID (e.g. *Vmn1r90/168/177*) or in the format “basalgeneID” if the number of gene IDs exceeded three (the lowest gene ID number was used). Thus V1R orthologs were identified using both sequence homology and phylogenetic relationships among receptors for all 6 species. Additionally, V1Rs that have evidence for gene losses in the house mouse (and corresponding pseudogenes) could not be annotated with the pseudogene ID, as often there are functional V1Rs with the same ID number. As a result, all gene losses in the house mouse detected in multiple sequenced mouse species are provided a new gene ID that does not overlap with any existing gene numbers. The annotated V1R coding sequences for all 5 sequenced *Mus* species sequenced are provided in Additional Files 4, 5, 6, 7 and 8.

### Phylogenetic analysis

All V1R peptide sequences for all 6 *Mus* species were aligned in MAFFT v. 7 (Additional File 9) [78]. Phylogenetic relationships were inferred using RAxML v. 8 to generate a maximum likelihood gene trees (based on peptide sequences) with 1000 replicates of bootstrapping (Additional File 1) [79]. Trees were visualized in FigTree v1.4.3 [80]. A few traditionally separated clades were combined due to a lack of clear clade separation when viewed across all 6 *Mus* species (clades: A/B and J/K) [81].

### Estimating gene duplicates

The well-characterized V1R repertoire of the reference genome was used to make estimates about which sequenced V1R transcripts are putative transcript variants or gene duplications within a given ortholog group (Additional File 2). Out of all the V1R transcripts in the reference, 55% code for the same peptide sequence, while 9.4% encode different peptides. Among the transcript variants encoding different peptides, sequence variation consists of either shorter sequences (i.e. only one exon is present) or variation at the ends of the transcript surrounding regions with gaps in pairwise alignments. We classified any V1R transcripts that (1) code for the same peptide, (2) whose variation consists of shortened coding sequences (i.e. only one exon is present), (3) whose variation falls at the ends of the transcript, or (4) whose variation falls near gaps in pairwise alignments, as putative transcript variants. Transcripts classified as putative gene duplicates were only those transcripts with at least one amino acid change central to the transcript, and not surrounded by gaps in pairwise alignments. This was only observed among different genes in the reference, never among transcript variants. Pairwise alignments were performed using EMBOSS Needle [82]. Due to the dynamic nature of V1R evolution and the incomplete V1R repertoires recovered for each species, duplications aren’t examined based on whether they are shared among species or are species-specific. Rather, duplications within ortholog groups are treated independently for each species.

### Positive selection analysis

We performed selection analyses on orthogroups containing at least 4 orthologs and/or paralogs. To test for whether genes were under positive selection we used the adaptive branch-site relative effects-likelihood (aBSREL) model based on dN/dS estimates as implemented in the software HyPhy [58, 83]. aBSREL was run on each orthogroup of sufficient size to identify branches with evidence of positive selection. *P* values from each aBSREL run were corrected for multiple testing using a false-discovery rate of 5%. After correction P values ≤0.05 were considered evidence for positive selection.

### Transmembrane helix prediction and mutation analysis

We focused on a two clade J/K receptors (*Vmn1r85* and *Vmn1r89*) for a more detailed examination of the amino acid changes across species, as clade J/K is a small highly orthologous clade, in which 2/4 receptor groups have the most well-supported evidence for de-orphanization [40, 49]. MAFFT v. 7 was used to align the orthologous receptor sequences [78]. TMHMM v. 2.0 was used to predict the locations of transmembrane helices [84], and which V1R protein regions are intracellular versus extracellular. For both *Vmn1r85* and *Vmn1r89* receptor groups, transcripts were aligned, transmembrane regions predicted, and sites with amino acid differences were identified across species (Figures S4 & S5). These amino acid differences are shown in protein schematics for each receptor (Figure S6). Four types of amino acid sites were characterized: (1) sites with an amino acid difference present in a single species, (2) sites with distinct amino acid differences in two different species, (3) sites with an amino acid difference shared between two to three species, (4) highly variable sites, in which amino acid differences suggest dynamism across the phylogenetic history of the genus (Figure S6). To examine *Vmn1r89* amino acid differences across species, the short transcriptional variants were excluded (Figure S4).

## Supplementary information

**Additional file 1. **Maximum likelihood gene tree of all 6 *Mus* species’ V1R peptide sequences. (txt)

**Additional file 2. **Categorization of V1R transcripts as either putative transcript variants or gene duplicates for the 5 non-commensal *Mus* species sequenced.

**Additional file 3: Table S1**. V1R orthogroup branches under positive selection across clades indicated by species. **Table S2.** V1Rs with evidence for positive selection, conservation (orthology and sequence identity) or gene expansions (across or within species). **Figure S1.** Number of V1R genes with splice variants in *M. m. domesticus* and *M. spretus*. **Figure S2**. Novel clade “N”: *Vmn1r248* and *Vmn1r249.***Figure S3. Left:** V1R gene tree clades E and F, displaying long internal branch lengths and short terminal branch lengths. ***Right***: Multiple alignments of *Vmn1r69* and *Vmn1r71* peptide sequences. **Figure S4.** Alignment and pairwise comparisons of *Vmn1r89* peptide sequences. **Figure S5.** Alignment and pairwise comparisons of *Vmn1r85* peptide sequences. **Figure S6.** Amino acid site changes in clade J/K receptors: *Vmn1r89* and *Vmn1r85*. (docx)

**Additional file 4. ***M. spicilegus* coding sequences of *V1r* genes expressed in the VNO. Gene annotations are abbreviated and contain species identifier “z”: *Vmn1r137* as 137-z. (fasta).

**Additional file 5. ***M. macedonicus* coding sequences of *V1r* genes expressed in the VNO. Gene annotations are abbreviated and contain species identifier “x”: *Vmn1r137* as 137-x. (fasta).

**Additional file 6. ***M. spretus* coding sequences of *V1r* genes expressed in the VNO. Gene annotations are abbreviated and contain species identifier “s”: *Vmn1r137* as 137-s. (fasta).

**Additional file 7. ***M. caroli* coding sequences of *V1r* genes expressed in the VNO. Gene annotations are abbreviated and contain species identifier “c”: *Vmn1r137* as 137-c. (fasta).

**Additional file 8. ***M. pahari* coding sequences of *V1r* genes expressed in the VNO. Gene annotations are abbreviated and contain species identifier “p”: *Vmn1r137* as 137-p. (fasta).

**Additional File 9. **Multiple sequence alignment of all 6 *Mus* species’ V1R peptide sequences. (fasta)

## Data Availability

All transcriptome sequencing data generated in this study are available in the NCBI Short Read Archive under BioProject PRJNA596328. The sequences and datasets used in this study are included in the manuscript and its additional files.

## Abbreviations

OR: Main olfactory receptor
VR: Vomeronasal receptor
V1R: Vomeronasal type 1 receptor
V2R: Vomeronasal type 2 receptor
VNO: Vomeronasal organ
Orthogroup: Orthologous receptor group
