# Supplementary material for: Distinct evolutionary trajectories of V1R clades across mouse species
Source: BMC Evol Biol. 2020 Aug 8;20:99. doi: 10.1186/s12862-020-01662-z (PMC7414754; doi:10.1186/s12862-020-01662-z)

**Table S1. V1R orthogroup branches under positive selection across clades indicated by species.** Species specified with strain names: *M. m. domesticus (*C57BL/6J) *M. spicilegus* (ZRU), *M. macedonicus* (XBS), *M. spretus* (SFM), *M. caroli* (CAR), and *M. pahari* (PAH). Terminal branches correspond to genes. Internal branches or nodes correspond to deeper branches in the orthogroup, which can contain multiple species.

| CLADE | TERMINAL Branch Species | INTERNAL Branch Node Species | All Species |
| --- | --- | --- | --- |
| A/B | **XBS** (Vmn1r47/48), **C57BL/6J** (Vmn1r52) | **XBS** (Vmn1r45),  **C57BL/6J & SFM** (Vmn1r47/48) | **C57BL/6J, XBS, SFM** |
| C | **ZRU** (Vmn1r11), **SFM** (Vmn1r34),  **PAH** (Vmn1r21, Vmn1r25/30) | **PAH, XBS & ZRU** (Vmn1r38/39) | **ZRU, XBS, SFM, PAH** |
| D | **ZRU** (Vmn1r60/61), **XBS** (Vmn1r172/173/174),  **SFM** (Vmn1r183), **CAR** (V1rd19, Vmn1r172/173/174) | NA | **ZRU, XBS, SFM, CAR** |
| E | **XBS** (Vmn1r241) & **ZRU** (Vmn1r241) | NA | **XBS, ZRU** |
| F | **CAR** & **SFM** (Vmn1r235) | NA | **SFM, CAR** |
| G | **ZRU** (Vmn1r74, Vmn1r76, Vmn1r242),  **SFM** (Vmn1r81), **CAR** (Vmn1r83), **PAH** (Vmn1r242) | **SFM, CAR & PAH** (Vmn1r242) | **ZRU, SFM, CAR, PAH** |
| H | **SFM** (Vmn1r205), **CAR** (Vmn1r206/209, 222),  **XBS** (Vmn1r247) | NA | **XBS, SFM, CAR** |
| I | **SFM** (Vmn1r192), **ZRU** (Vmn1r193) | NA | **ZRU, SFM** |
| J/K | NA | **Ref, ZRU, XSB & SFM** (Vmn1r85) | **Ref, ZRU, XSB, SFM** |
| L | NA | NA | **NA** |
| N | NA | NA | **NA** |

**Table S2. V1Rs with evidence for positive selection, conservation (orthology and sequence identity) or gene expansions (across or within species).** Species indicated with letters: *M. m. domesticus* (m), *M. spicilegus* (z), *M. macedonicus* (x), *M. spretus* (s), *M. caroli* (c), and *M. pahari* (p).

| CLADE | Clade-Wide Evolutionary Pattern | Positive Selection Detected | Conserved Sequence Identity | Highest Orthology | Multi-Species  Expansions | Species-Specific  Expansions | House Mouse Specific Expansions |
| --- | --- | --- | --- | --- | --- | --- | --- |
| A/B | Somewhat Dynamic | Vmn1r45, Vmn1r47/48, Vmn1r52 | Vmn1r51 | Vmn1r1, Vmn1r40, Vmn1r44, Vmn1r47/48 | Vmn1r45, Vmn1r47/48 | Vmn1r44 (p), Vmn1r47/48 (s), Vmn1r50 (s) | Vmn1r52/V1ra8 |
| C | Dynamic | Vmn1r11, Vmn1r21, Vmn1r25/30, Vmn1r38/39 | --- | Vmn1r11, Vmn1r13, Vmn1r19, Vmn1r20/27, Vmn1r24, Vmn1r25/30, Vmn1r26, Vmn1r38/39 | Vmn1r19, Vmn1r38/39, Vmn1r7/8, Vmn1r9/10 | Vmn1r19 (c), Vmn1r33 (s), Vmn1r36/37 (p),  Vmn1r38/39 (z, s, p), Vmn1r7/8 (p) | --- |
| D | Dynamic | Vmn1r60/61, Vmn1r172/173/174, Vmn1r183, V1rd19 | --- | Vmn1r172/173/174, Vmn1r179, V1rd19 | Vmn1r168/177, Vmn1r180 | Vmn1r167 (z),  Vmn1r90 (s) | Vmn1r172/173/174, Vmn1r60/61, Vmn1r62/186, Vmn1r63/187, Vmn1r100/148, Vmn1r170/175, Vmn1r56, Vmn1r57, Vmn1r91, Vmn1r93-95, Vmn1r101, Vmn1r103, Vmn1r104, Vmn1r107, Vmn1r111-132, Vmn1r135, Vmn1r137-139, Vmn1r142, Vmn1r143, Vmn1r149, Vmn1r151, Vmn1r152, Vmn1r155, Vmn1r157-160, Vmn1r163, Vmn1r165, Vmn1r166, Vmn1r171, Vmn1r100/148 |
| E | Conserved | Vmn1r241 | Vmn1r224, Vmn1r227 | Vmn1r224, Vmn1r227, Vmn1r230, Vmn1r231, Vmn1r68, Vmn1r71 | Vmn1r71 | --- | --- |
| F | Somewhat Conserved | Vmn1r235 | --- | Vmn1r236, Vmn1r237 | --- | --- | --- |
| G | Mixed: Conserved & Dynamic | Vmn1r74, Vmn1r76, Vmn1r81, Vmn1r83, Vmn1r242 | Vmn1r84 | Vmn1r73, Vmn1r74, Vmn1r79/2/3/238, Vmn1r84 | --- | --- | Vmn1r2/3/79/238 |
| H | Dynamic | Vmn1r205, Vmn1r206/209, Vmn1r247 | Vmn1r197 | Vmn1r196, Vmn1r206/209, Vmn1r214 | Vmn1r206/209,  Vmn1r247 | Vmn1r203 (s),  Vmn1r205 (s),  Vmn1r206/209 (c, p) | --- |
| I | Somewhat Dynamic | Vmn1r192, Vmn1r193 | Vmn1r192, Vmn1r218 | Vmn1r192, Vmn1r193, Vmn1r194, Vmn1r218 | Vmn1r247, Vmn1r192, Vmn1r193, Vmn1r202, Vmn1r216 | --- | --- |
| J/K | Mixed: Conserved & Dynamic | Vmn1r85 | --- | Vmn1r85, Vmn1r86, Vmn1r89 | Vmn1r86/88 | Vmn1r86/88 (c) | --- |
| L | Conserved | --- | Vmn1r70 | --- | --- | --- | --- |
| N | Mixed: Conserved & Dynamic | --- | --- | --- | --- | --- | --- |


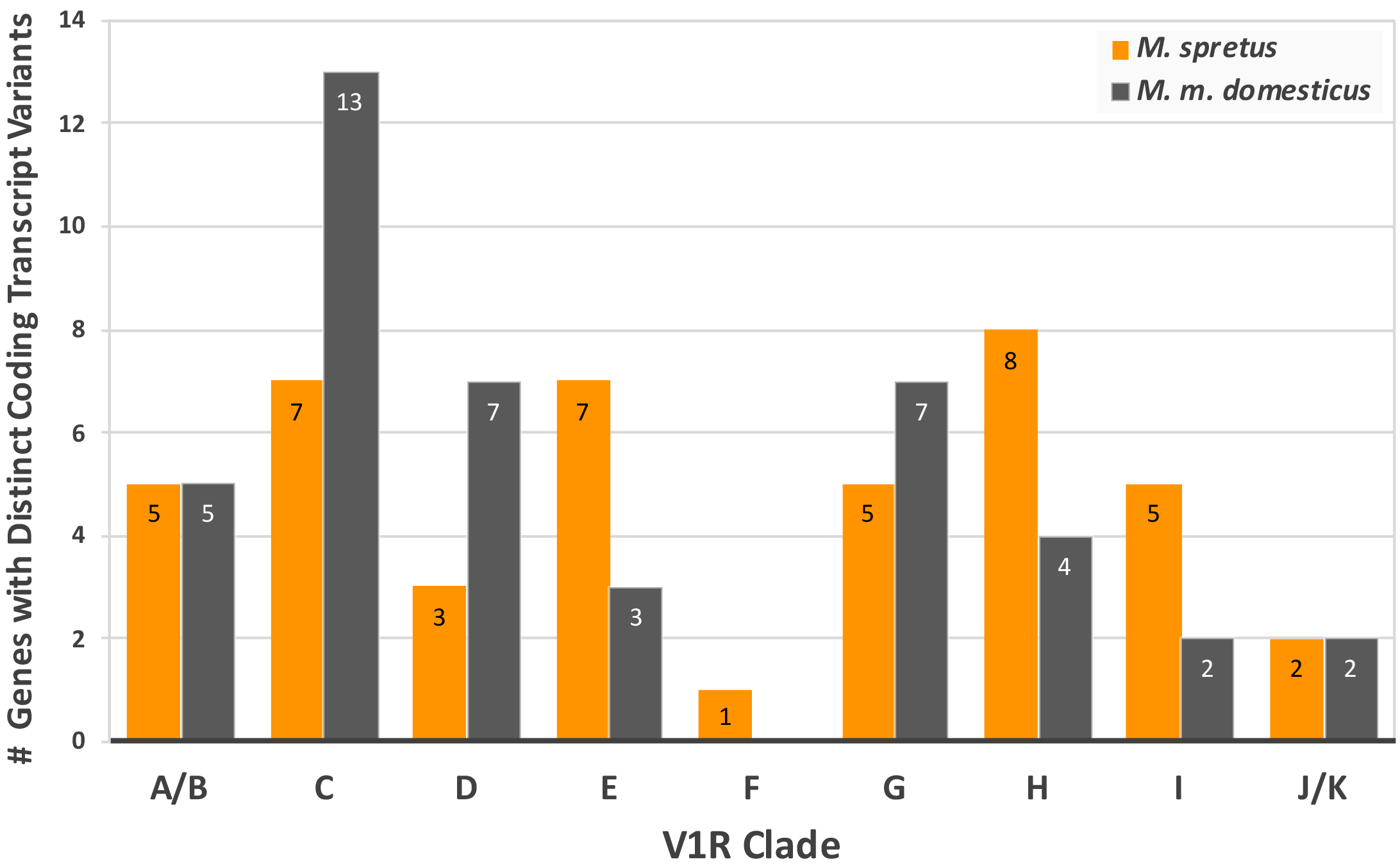


**Figure S1. Number of V1R genes with splice variants.** V1R genes by clade that express multiple coding transcripts with distinct peptide sequences in both the house mouse (grey) and *M. spretus* (orange).


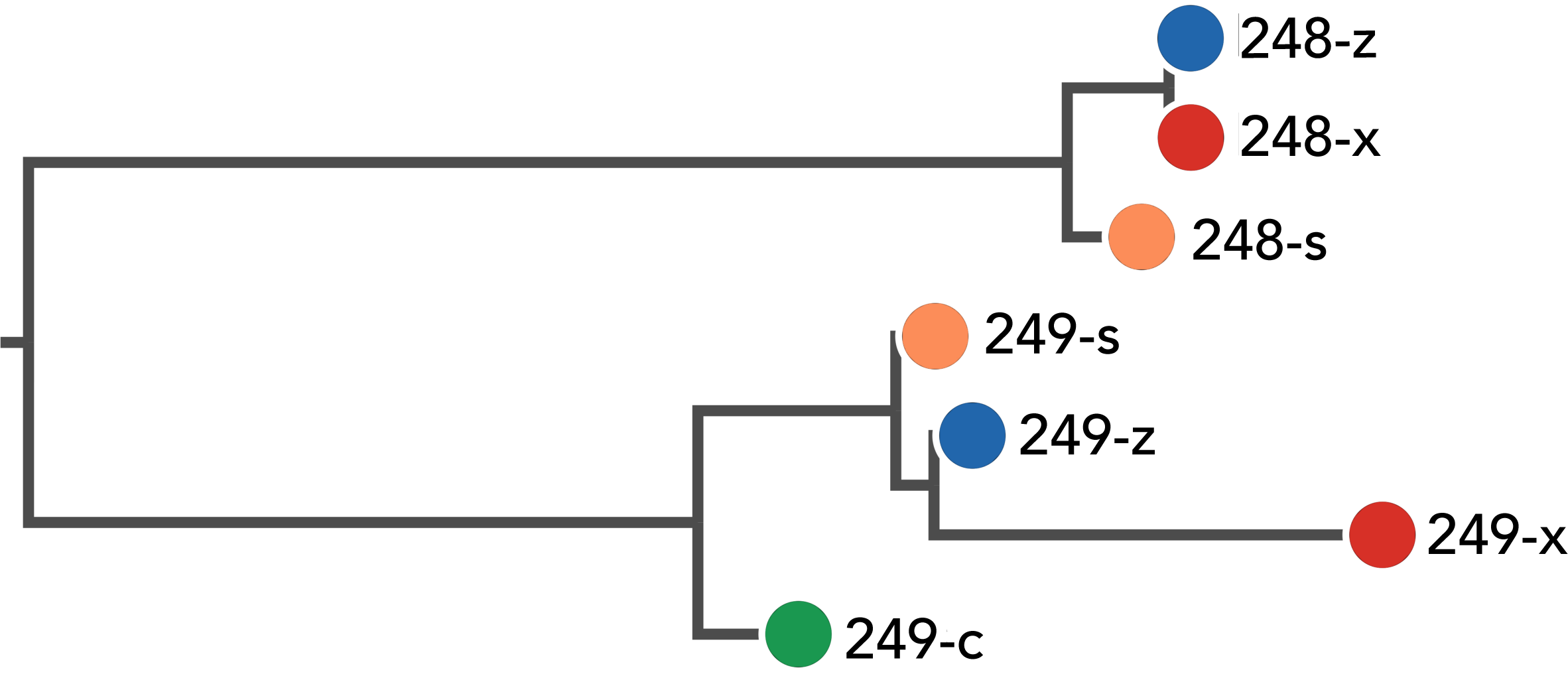


**Figure S2. Novel clade “N”.** V1R gene tree clade N with genes *Vmn1r248* and *Vmn1r249.* *Mus* species are indicated with letter abbrevations and colors (*M. spicilegus*: “z” and blue; *M. macedonics*: “x” and red; *M. spretus*: “s” and orange; and *M. caroli*: “c” and green).

**
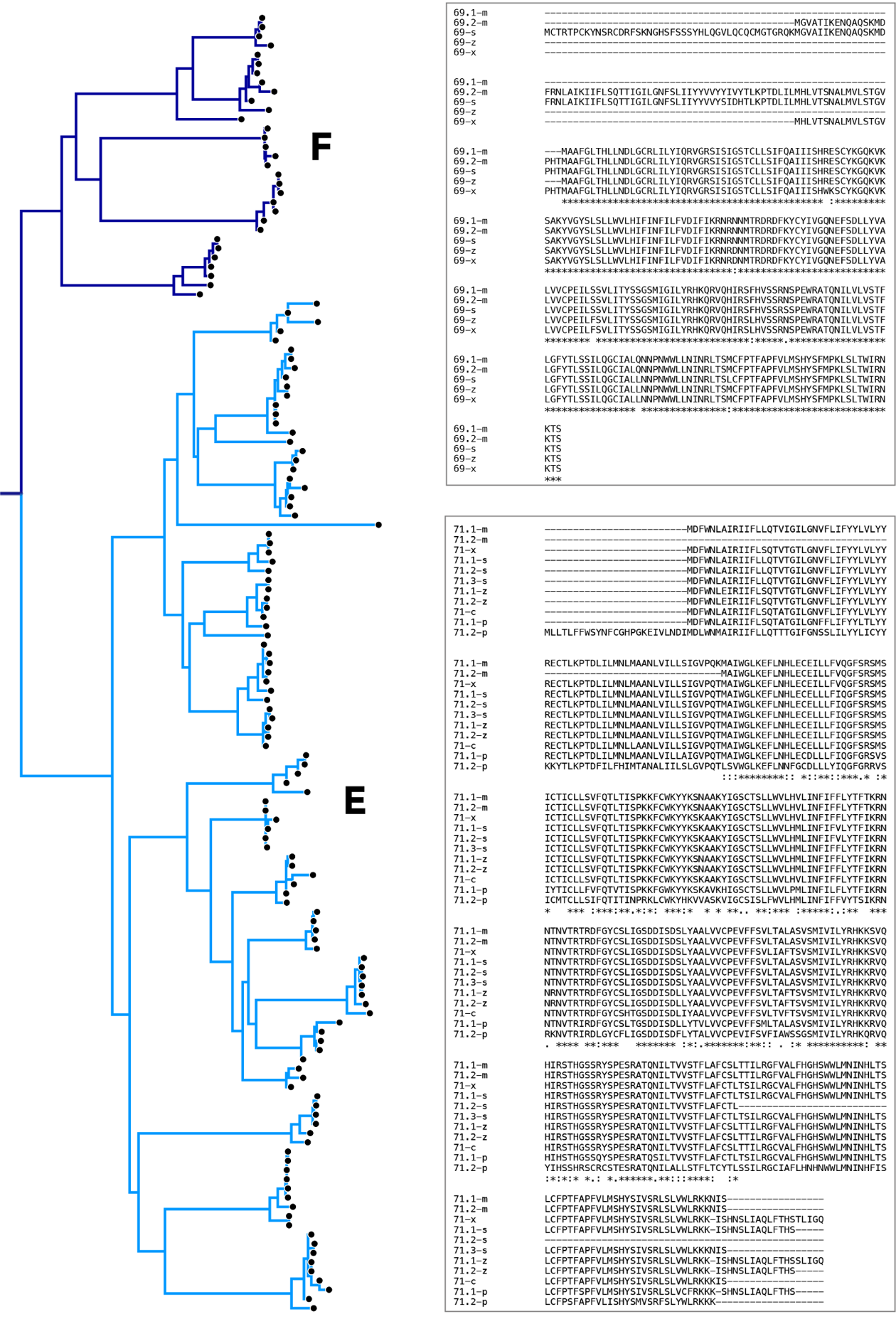
**

**Figure S3. *Left***: V1R gene tree clades E (light blue) and F (dark blue), displaying long internal branch lengths and short terminal branch lengths. ***Right***: Multiple alignment of *Vmn1r69* (top) and *Vmn1r71* (bottom) peptide sequences. *Mus* species indicated with letter abbrevations (*M. m. domesticus*: “m”, *M. spicilegus*: “z”, *M. macedonics*: “x”, *M. spretus*: “s”, *M. caroli*: “c” and *M. pahari*: “p”).


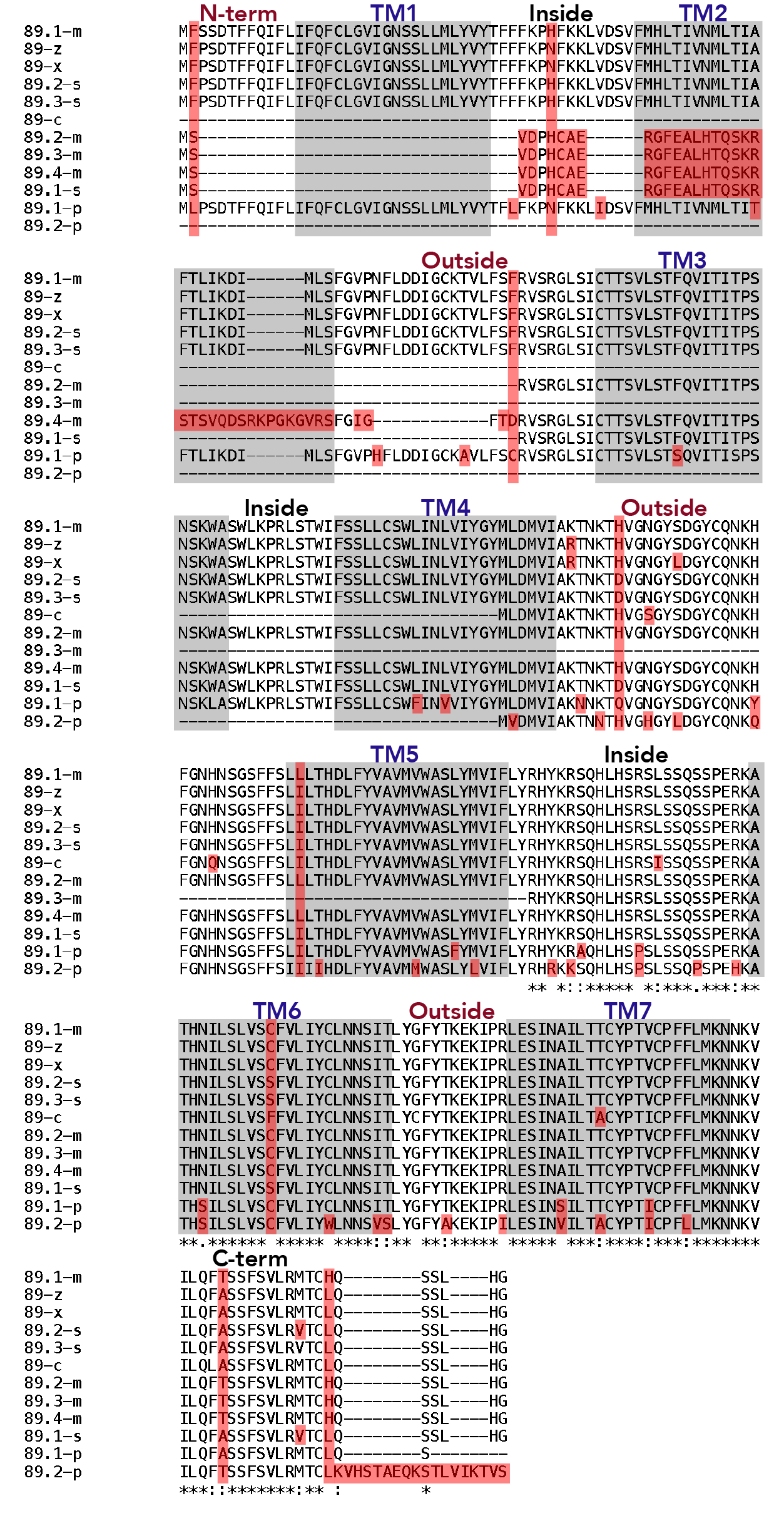

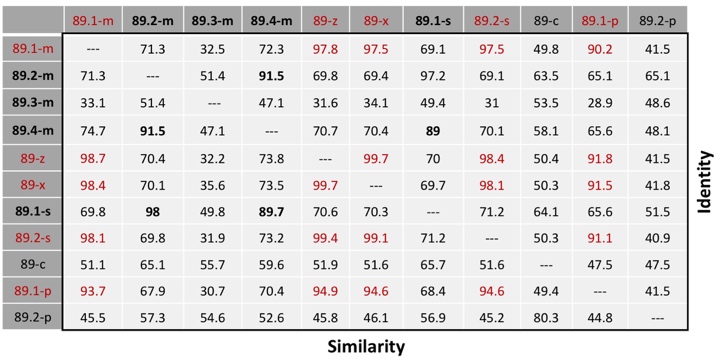


**Figure S4. Alignment and pairwise comparisons of *Vmn1r89* peptide sequences**. *Mus* species indicated with letter abbrevations (*M. m. domesticus*: “**m**”, *M. spicilegus*: “**z**”, *M. macedonicus*: “**x**”, *M. spretus*: “**s**”, *M. caroli*: “**c**” and *M. pahari*: “**p**”). ***Top***: **Sequence alignment.** Identical residues indicated by asterisks. Predicted transmembrane domains shown in grey. Amino acid differences indicated in red. ***Bottom***: **Pairwise similarities and identities.** Long variants of *Vmn1r89* indicated in red. Short transcript variants among *M. m. domesticus* and *M. spretus* indicated in bold. *Vmn1r89.3-s* is not shown, as it shares 100% amino acid identity with *Vmn1r89.2-s.*


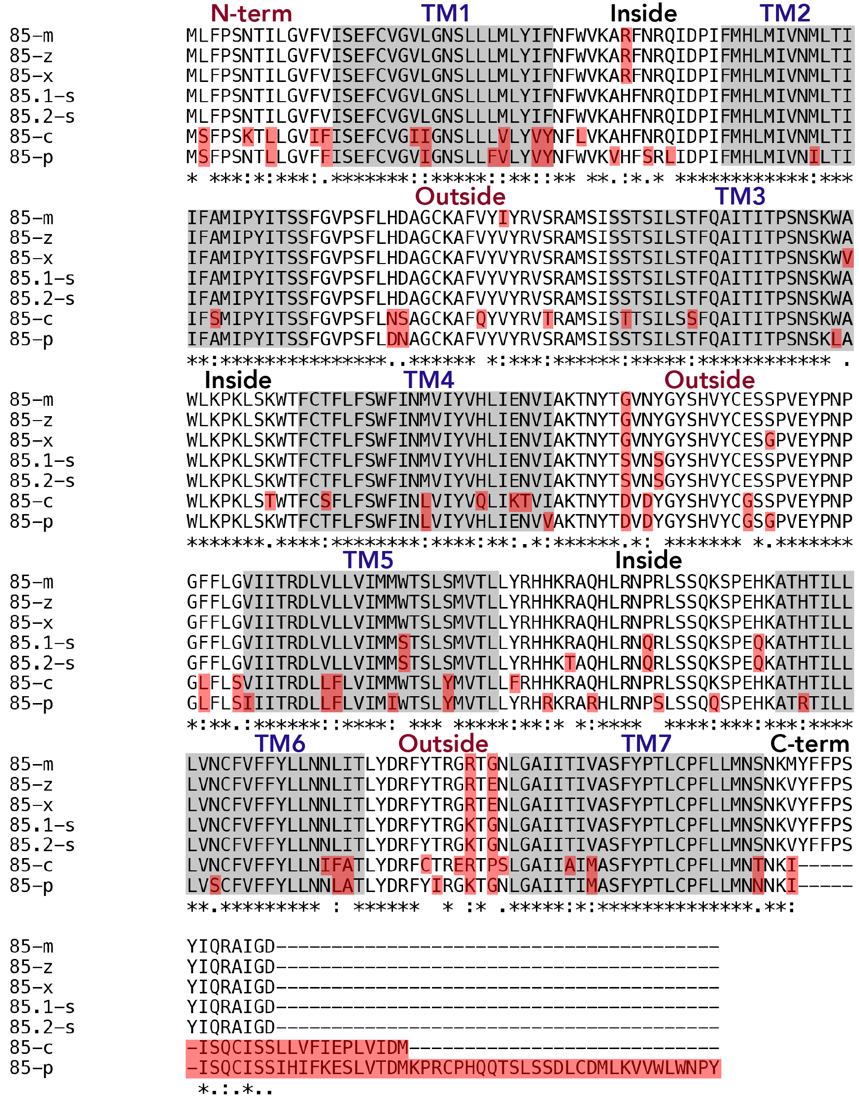

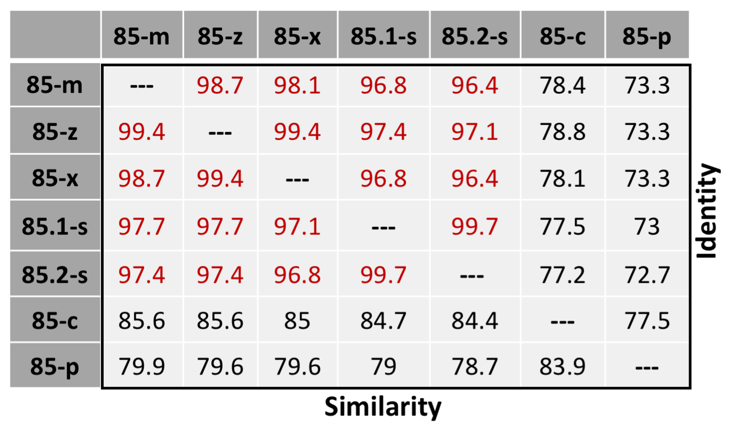


**Figure S5. Alignment and pairwise comparisons of *Vmn1r85* peptide sequences**. *Mus* species indicated with letter abbrevations (*M. m. domesticus*: “**m**”, *M. spicilegus*: “**z**”, *M. macedonicus*: “**x**”, *M. spretus*: “**s**”, *M. caroli*: “**c**” and *M. pahari*: “**p**”). ***Top***: **Sequence alignment.** Identical residues indicated by asterisks. Predicted transmembrane domains shown in grey. Amino acid differences indicated in red. ***Bottom***: ***Vmn1r85* amino acid pairwise similarities and identities.** Conserved sequences among species closely related to the house mouse indicated in red.

**Figure S6.** **Amino acid site changes in clade J/K receptors: Vmn1r89 and Vmn1r85**. The transmembrane protein structure of the V1Rs are depicted schematically. The location of all amino acid site changes in Vmn1r89 and Vmn1r85 across all 6 species are represented with stars. Mus species are indicated with colors and letters (M. m. domesticus: black; M. spicilegus: blue; M. macedonics: red; M. spretus: orange; M. caroli: green; M. pahari: purple). Stars with multiple colors indicate an amino acid change present in multiple species at that site. White stars indicate highly variable sites (several variable amino acid changes are present across multiple species). The percentage of amino acid site differences within each region (transmembrane, inside, or outside the membrane) across all species are indicated in table below. Short Vmn1r89 transcripts were excluded from this analysis.


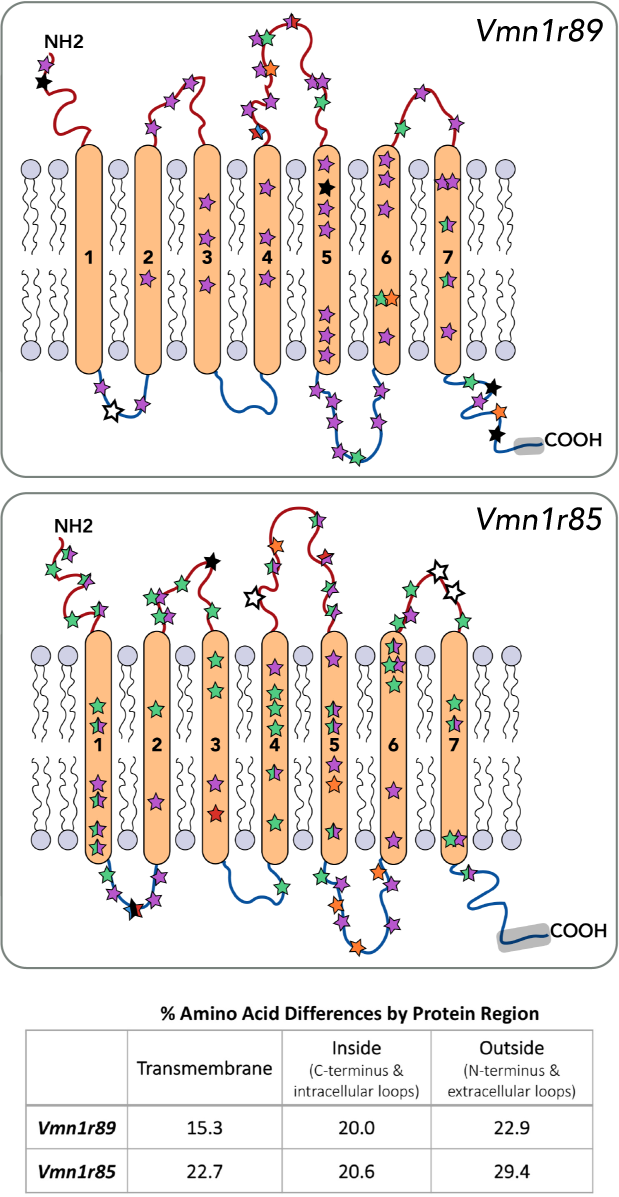

Supplement: Supplementary file 3 — Additional file 3: Table S1. V1R orthogroup branches under positive selection across clades indicated by species. Table S2. V1Rs with evidence for positive selection, conservation (orthology and sequence identity) or gene expansions (across or within species). Figure S1. Number of V1R genes with splice variants in M. m. domesticus and M. spretus. Figure S2. Novel clade “N”: Vmn1r248 and Vmn1r249.Figure S3. Left: V1R gene tree clades E and F, displaying long internal branch lengths and short terminal branch lengths. Right: Multiple alignments of Vmn1r69 and Vmn1r71 peptide sequences. Figure S4. Alignment and pairwise comparisons of Vmn1r89 peptide sequences. Figure S5. Alignment and pairwise comparisons of Vmn1r85 peptide sequences. Figure S6. Amino acid site changes in clade J/K receptors: Vmn1r89 and Vmn1r85. (docx) [file 12862_2020_1662_MOESM3_ESM.docx]
